# Burnout in Out-of-Hospital Emergency Healthcare Professionals: Systematic Review and Meta-Analysis

**DOI:** 10.3390/healthcare14213591

**Published:** 2026-10-23

**Authors:** María Jesús Rodríguez-Gómez, Sami Suleiman-Martos, José M. Gutiérrez-Pastor, Ismael Loonsri-Pizarro, Rubén García-Lara, Miguel Quesada-Caballero, Antonio M. Caballero-Mateos

**Affiliations:** 1 Loja Health Center, Granada-Metropolitan Health District, Andalusian Health Service, 18300 Granada, Spain; mrodrg19@correo.ugr.es; 2 Purullena Health Center, Northeast Health Area, Andalusian Health Service, Purullena, 18519 Granada, Spain; samism441992@gmail.com; 3 San Cecilio Clinical University Hospital, Andalusian Health Service, Av. del Conocimiento s/n, 18016 Granada, Spain; gutiforecast37@gmail.com; 4 Cabueñes University Hospital, Health Service of the Principality of Asturias Asturias, 33394 Gijón, Spain; ismaelloonsri@correo.ugr.es; 5 Íllora Health Center, Granada-Metropolitan, Health District, Andalusian Health Service, 18260 Granada, Spain; ruben.garcia.lara.sspa@juntadeandalucia.es; 6 Instituto de Investigación Biosanitaria (ibs.GRANADA), 18012 Granada, Spain; miguel.quesada.caballero.sspa@juntadeandalucia.es; 7 Albayda Health Center, Granada-Metropolitan Health District, Andalusian Health Service, 18015 Granada, Spain; 8 Department of Internal Medicine, Gastroenterology Section, Hospital Santa Ana, 18600 Motril, Spain

**Keywords:** burnout, emergency medical services, health personnel, occupational health, risk factors, meta-analysis

## Abstract

**Background/Objectives:** Burnout is an occupational phenomenon that particularly affects healthcare professionals exposed to sustained stress. In out-of-hospital emergency care, repeated exposure to critical incidents, violence, and demanding shift patterns may increase this risk. This review aimed to estimate the prevalence of burnout dimensions and identify factors associated with burnout among prehospital emergency care professionals. **Methods:** A systematic review and meta-analysis was conducted following PRISMA 2020 (PROSPERO CRD420261489417). CINAHL, LILACS, PubMed, Web of Science, and Scopus were searched in July 2026. Quantitative studies that measured burnout with the Maslach Burnout Inventory (MBI) or the Copenhagen Burnout Inventory (CBI) were included. Prevalences were pooled using random-effects models, and the certainty of the evidence was rated with GRADE. **Results:** Thirty-one studies (9926 unique participants) were included, 12 of which contributed to the meta-analyses. The pooled prevalence was 33% (95% CI 22–45%; I^2^ = 97.7%) for high emotional exhaustion, 37% (95% CI 26–48%; I^2^ = 97.1%) for high depersonalization and 27% (95% CI 17–38%; I^2^ = 97.4%) for low personal accomplishment, with overlapping confidence intervals and wide prediction intervals. The most consistent associated factors were exposure to workplace violence, lack of organizational and supervisor support, and demanding shift patterns; findings for professional role, gender, age, and years of experience were mixed. The certainty of the pooled estimates was very low. **Conclusions:** Around one in three prehospital professionals shows high levels in each burnout dimension, although estimates vary widely between settings. Organizational strategies focused on violence prevention, supervisory support, and early detection are needed.

## 1. Introduction

Burnout syndrome (BS) has become a silent threat in healthcare settings. In 2019, the World Health Organization (WHO) included this syndrome in the 11th revision of the International Classification of Diseases (ICD-11) as an occupational phenomenon resulting from chronic workplace stress. It refers specifically to phenomena in the occupational context and is not classified as a medical condition [1,2]. BS is a psychological syndrome composed of three dimensions: emotional exhaustion (EE), depersonalization (D), and reduced personal accomplishment (PA). EE refers to feeling overextended and depleted of emotional and physical resources. D refers to a negative, callous, or excessively detached response to various aspects of the job, and reduced PA refers to feelings of incompetence and a lack of achievement and productivity at work [3].

According to the model of Maslach and Leiter [4], burnout develops progressively from a mismatch between individuals and their work environment in six areas: workload, control, reward, community, fairness, and values. From this perspective, burnout is not only an individual response to stress but also a relational and organizational phenomenon, with direct implications for the quality of care, patient safety, and the sustainability of the healthcare system.

There is no consensus on the prevalence of burnout, but it is recognized as an increasingly common problem [5]. Healthcare professionals are among the occupational groups with the highest levels of work-related stress and burnout, a trend that became more pronounced during the COVID-19 pandemic, when its contributing factors were exacerbated [6,7,8]. The consequences of burnout extend beyond the health and well-being of workers to the care they provide: the Minimizing Error, Maximizing Outcome (MEMO) study showed that stressed and dissatisfied professionals are more likely to make errors and to provide suboptimal care [9,10].

Burnout also has economic consequences for healthcare institutions [11,12]. It has been associated with increased absenteeism, higher staff turnover, lower productivity, and greater use of healthcare resources, all of which considerably increase healthcare costs [12,13,14].

Prehospital emergency care differs clearly from hospital practice. Multidisciplinary teams of physicians, nurses, paramedics, and emergency medical technicians provide care in uncontrolled and often unsafe environments, such as roads, homes, or public spaces, with limited material resources and little immediate back-up, and they must make time-critical decisions with incomplete information [15]. These professionals are repeatedly exposed to critical incidents, such as serious trauma or the death of children, which can affect their mental health and emotional well-being [15,16], and to verbal and physical violence from patients and bystanders [17,18,19].

In addition, work in emergency medical services is organized in long or irregular shifts, frequently of 24 h or more, which are associated with poor sleep and fatigue. In a multicenter study, more than half of emergency medical services providers reported fatigue, which was associated with higher odds of injury, medical errors, or adverse events and safety-compromising behaviors [20]. The demand generated by non-urgent calls and the perceived lack of management support have also been described as stressors specific to ambulance work [21,22,23].

These demands may influence not only the overall level of burnout but also the profile of its dimensions: repeated exposure to critical incidents and long shifts may deplete emotional resources (EE), distancing from patients may act as a defensive response to violence and suffering (D), and limited recognition and support may reduce the sense of professional efficacy (PA). Therefore, findings from hospital-based professionals cannot be directly extrapolated to the prehospital setting.

Although burnout has been widely studied in healthcare professionals [24,25,26,27], previous reviews have addressed prehospital personnel only partially. Reardon et al. identified only five prevalence studies in paramedics, with prevalences ranging from 16% to 56%, and could not pool them because of the heterogeneity of instruments [28]. Other reviews of prehospital services have focused on the qualitative experience of ambulance work [15] or on job satisfaction and work engagement [29], and a recent meta-analysis estimated an overall burnout prevalence of 40% (95% CI 33–49%; prediction interval 9–82%) among emergency medical services personnel [30]. However, no previous review has jointly synthesized the dimension-specific prevalence of burnout, measured with standardized instruments, and the sociodemographic, occupational, violence-related, and organizational factors associated with burnout exclusively in prehospital professionals, including the Spanish- and Portuguese-language literature. Therefore, the aim of this systematic review and meta-analysis, conducted in accordance with the PRISMA guidelines [31], was to estimate the prevalence of high EE, high D, and low PA and to identify the factors associated with burnout among out-of-hospital emergency healthcare professionals.

## 2. Materials and Methods

### 2.1. Protocol and Registration

This systematic review and meta-analysis was conducted and reported according to the Preferred Reporting Items for Systematic Reviews and Meta-Analyses (PRISMA) 2020 statement [31] (Table S1). The protocol was registered in PROSPERO (CRD420261489417).

The following deviations from the registered protocol occurred: (i) risk of bias was assessed with the JBI checklist for prevalence studies instead of the CASPe and SIGN tools, because the JBI checklist is specific to prevalence data; (ii) studies that used the CBI were synthesized narratively rather than pooled, because the CBI does not measure the three MBI dimensions; (iii) studies in which a minority of non-eligible staff could not be separated were retained and examined in sensitivity analyses; and (iv) prediction intervals, sensitivity and subgroup analyses, and the GRADE assessment of certainty were added during peer review.

### 2.2. Eligibility Criteria

Studies were included if they (1) were quantitative observational studies (cross-sectional or longitudinal studies, or the quantitative component of mixed-methods studies) that assessed burnout in healthcare professionals (physicians, nurses, paramedics and emergency medical technicians) working in prehospital emergency and urgent care; (2) measured burnout with any version of the Maslach Burnout Inventory (MBI) or with the Copenhagen Burnout Inventory (CBI); and (3) were published in English, Spanish or Portuguese, with no date restriction. Studies of students or trainees, volunteers or non-healthcare personnel and doctoral theses were excluded. Studies with mixed samples of prehospital and hospital professionals were excluded unless data for prehospital professionals were reported separately, in which case only these data were extracted. Studies in which non-eligible staff (e.g., ambulance drivers, dispatchers, or volunteers) were a minority of the sample and could not be separated were retained and examined in sensitivity analyses, whereas studies in which non-eligible personnel were the majority were excluded.

The MBI and CBI were selected because they are the most widely used and validated instruments for measuring burnout in healthcare professionals. Because they operationalize burnout differently (the MBI as EE, D and PA; the CBI as personal, work-related, and patient-related burnout), studies that used the CBI were synthesized narratively and were not pooled with the MBI dimensions.

### 2.3. Information Sources and Search Strategy

CINAHL, LILACS, PubMed, Web of Science, and Scopus were searched in July 2026. The search combined terms for burnout, prehospital emergency professionals, and the MBI/CBI and was adapted to each database with controlled vocabulary and field tags; the complete database-specific strategies are provided in Table S2. Backward (reference lists) and forward (the “Cited by” functions of Web of Science and Scopus) citation searching of the included studies was also performed. Because requiring instrument terms in the search string could miss eligible studies that mention the instrument only in the full text, citation searching was used to mitigate this risk, which is acknowledged as a limitation.

### 2.4. Study Eligibility

**Figure 1:**
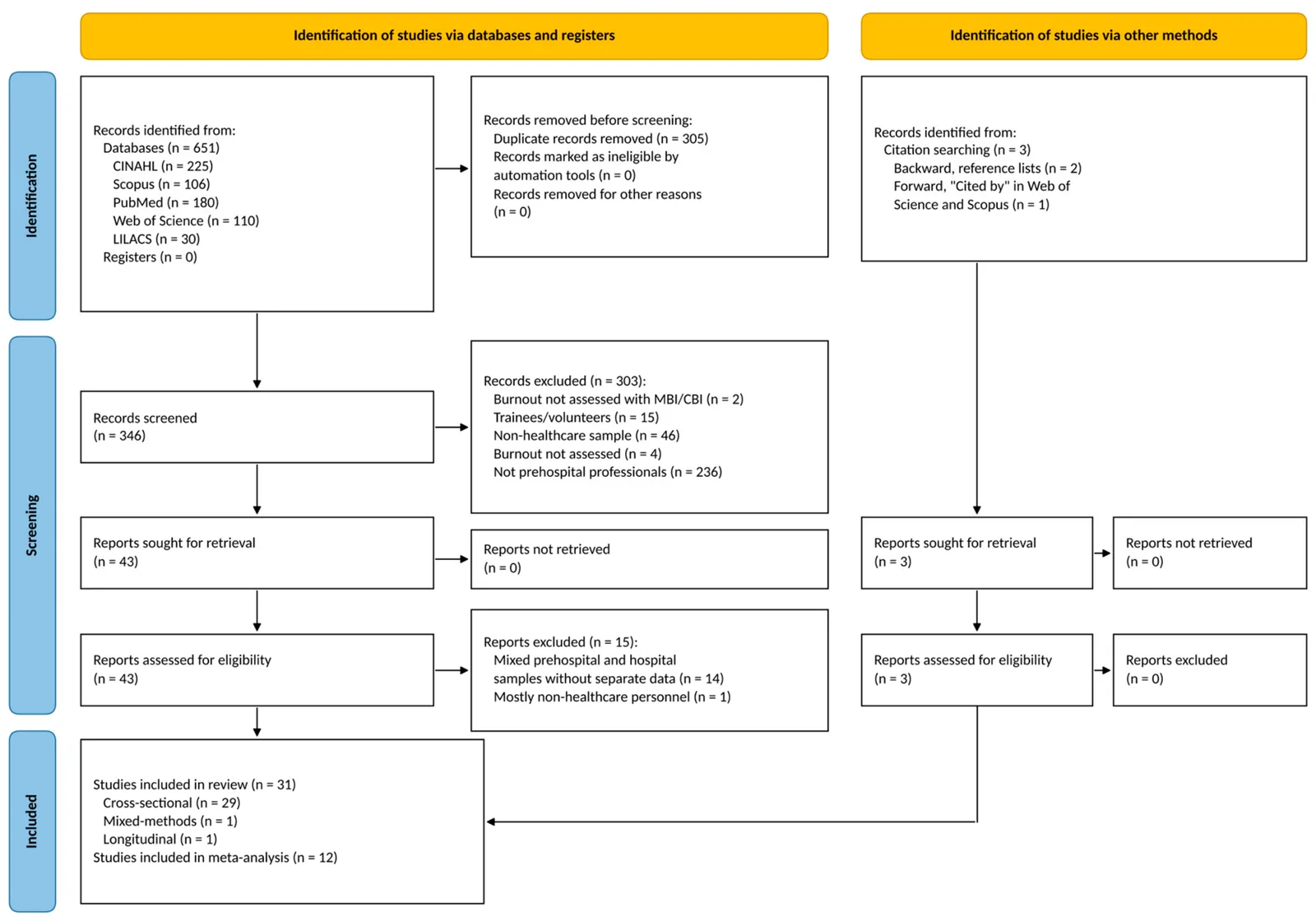
PRISMA 2020 flow diagram of the study selection process.

After removal of duplicates, two reviewers independently screened titles and abstracts and then assessed the full texts of potentially eligible studies. Disagreements were resolved by discussion and, when necessary, by a third reviewer. Records were managed in Zotero, and screening decisions were recorded in Microsoft Excel. The selection process is summarized in the PRISMA flow diagram (Figure 1).

### 2.5. Data Extraction

Data were extracted by one reviewer using a standardized form piloted in Microsoft Excel and were checked by a second reviewer. The following data were extracted: first author, year, country, design, sample size and professional group, instrument and version, cut-off points, prevalence of overall burnout and of each dimension (or mean and standard deviation values when prevalences were not reported), and associated factors. Discrepancies between reviewers were resolved by discussion or, if necessary, by a third reviewer. As a final check, all extracted data were re-verified against the original articles.

### 2.6. Risk of Bias Assessment

Risk of bias was assessed independently by two reviewers using the Joanna Briggs Institute (JBI) Critical Appraisal Checklist for Studies Reporting Prevalence Data. This comprises nine items addressing the sampling frame, sampling method, sample size, description of subjects and setting, coverage of the sample, validity and reliability of the measurement, statistical analysis, and response rate [32]. Disagreements were resolved by consensus. The assessment of each study is reported in Table S3.

### 2.7. Outcome Definitions

The outcomes were the proportions of professionals with high EE, high D, and low PA according to the MBI cut-offs used in each study. Because studies applied normative, country-specific, or sample-based cut-offs, the categories were accepted as defined by the authors, and the direction of each category was checked in every study so that “low PA” always corresponded to a low PA score (i.e., the burnout-indicative category). A study was included in the meta-analysis of a given dimension when it reported, or allowed the calculation of, the proportion of participants in the burnout-indicative category. Studies were not pooled when they reported only mean, median, or total scores [18,19,33,34,35,36,37,38,39,40,41], used sample-based tertile cut-offs with overlapping category ranges [42], did not report dimension-specific data [43,44] (for [43], only the 181 full-time prehospital professionals, reported separately, were extracted), used the CBI [21,22,45], or used the MBI-General Survey, whose dimensions are not equivalent [46]. Roth et al. [47] was not pooled because its sample came from the same 2015 EMSiG survey as that of Baier et al. [48]. When a study reported the inverse dimension (e.g., “professional incompetence”), its burnout-indicative category was used as low PA [49]. When non-eligible staff were reported separately, only eligible professionals were extracted (ambulance drivers were excluded from [49]); when they were not reported separately [50,51], the study was retained, and its influence was examined in a sensitivity analysis.

### 2.8. Statistical Analysis

Prevalences were pooled with random-effects models using the Freeman–Tukey double arcsine transformation, with between-study variance estimated by the DerSimonian–Laird method and back-transformation based on the harmonic mean of the sample sizes. Exact (Clopper–Pearson) 95% confidence intervals (CIs) were calculated for individual studies. Heterogeneity was assessed with Cochran’s Q, I^2^, and τ^2^, and 95% prediction intervals were calculated.

Sensitivity analyses included a leave-one-out analysis and the exclusion of studies that included non-eligible staff not reported separately [50,51]. An exploratory subgroup analysis by region (Europe vs. other regions) was performed with the Q-between test; because only two pooled studies collected data during the COVID-19 pandemic, they were compared descriptively with those conducted before the pandemic. Publication bias was assessed with Egger’s regression test, interpreted with caution given the small number of pooled studies. Analyses were performed with StatsDirect (version 3, StatsDirect Ltd., Cambridge, UK). Studies not eligible for pooling were synthesized narratively. The certainty of each pooled estimate was rated with the GRADE approach adapted for estimates of prevalence and event rates [52], starting at high certainty and downgrading for risk of bias, inconsistency, imprecision, indirectness, and publication bias.

## 3. Results

### 3.1. Study Selection

The search identified 651 records (CINAHL 225, Scopus 106, PubMed 180, Web of Science 110, and LILACS 30). After removal of 305 duplicates, 346 records were screened, and 303 were excluded. Of the 43 full texts assessed, 15 were excluded: 14 because they included mixed prehospital and hospital samples without separate data and one because most of its participants were police officers. Three additional studies were identified through backward (n = 2) and forward (n = 1) citation searching. In total, 31 studies were included in the review and 12 in the meta-analyses (Figure 1).

### 3.2. Characteristics of the Included Studies

The 31 included studies comprised 10,950 participants, counting for each study the sample shown in Table 1 (the baseline sample of [46], the 441 participants of [17], the 700 participants of [44] and only the 181 full-time prehospital professionals of [43]); because the samples of [47,48] came from the same survey, the number of unique participants was 9926. Twenty-nine studies were cross-sectional (one of them correlational [33]), one was a mixed-methods survey [22], and one was longitudinal [46]. The MBI was the most frequently used instrument, mainly the MBI-Human Services Survey; the MBI-General Survey [34,43,46], an abbreviated 12-item MBI [41], and the CBI [21,22,45] were also used, with normative, country-specific, or sample-based cut-offs. Ten studies were conducted in Europe [17,22,34,36,37,46,47,48,53,54], six in the Americas (USA [21,51], Canada [55], Mexico [50] and Brazil [49,56]), thirteen in Asia [18,19,33,35,38,39,40,42,43,44,45,57,58], one in Africa [59] and one in Oceania [41]. Among the studies that reported the proportion of participants with overall burnout, as defined by each study (at risk of burnout, high EE and/or D, or a CBI score ≥ 50), this proportion ranged from 8.6% [46] to 76.3% [56].

Twelve studies [22,33,34,35,40,42,43,44,45,47,50,53] were published between 2022 and 2026, which reflects a growing interest in this topic. The characteristics of each study are summarized in Table 1.

### 3.3. Risk of Bias

According to the JBI checklist, 10 of the 31 studies met at least seven of the nine criteria (median 6; range 1–9) (Table S3). The most frequent shortcomings were non-probabilistic sampling (item 2; 16 studies), an insufficient or unjustified sample size (item 3; 14 studies), incomplete coverage of the identified sample (item 5; 24 studies), and a low or unreported response rate (item 9; 24 studies). All studies used validated instruments (the MBI or CBI; item 7). No study was excluded on the basis of its methodological quality.

### 3.4. Pooled Prevalence of Burnout Dimensions

**Figure 2:**
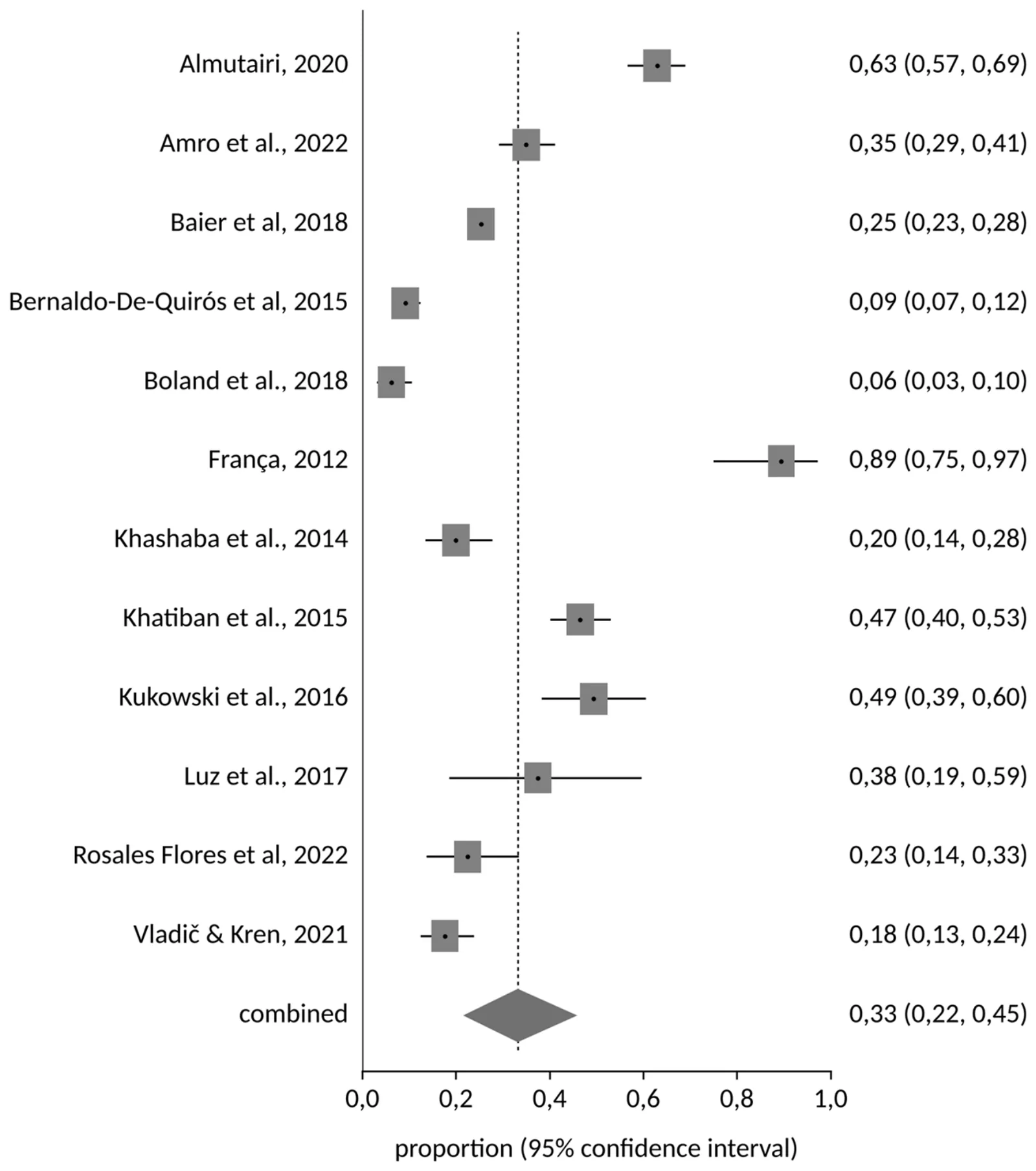
Forest plot of the pooled prevalence of high emotional exhaustion (random-effects model).

**Figure 3:**
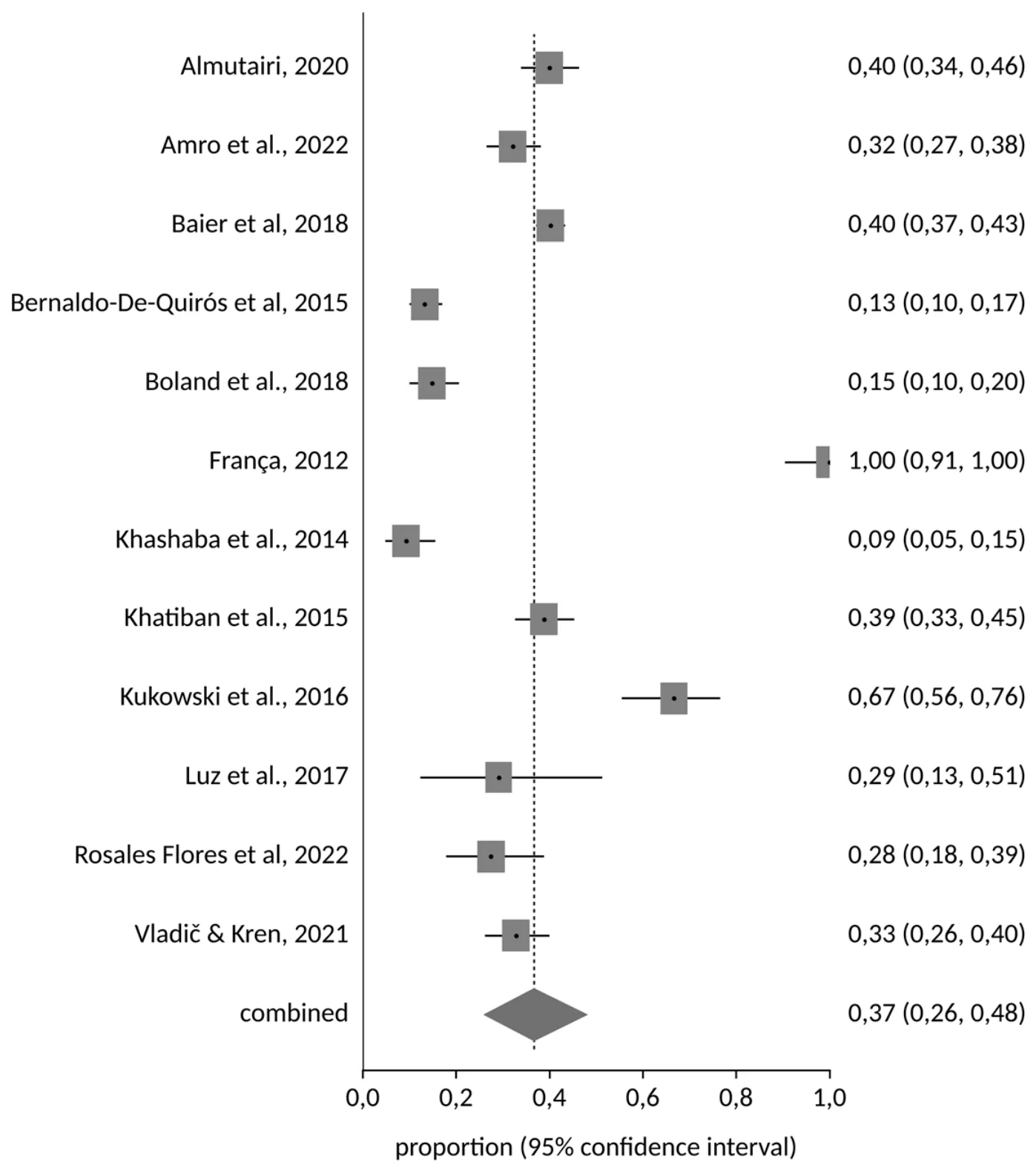
Forest plot of the pooled prevalence of high depersonalization (random-effects model).

**Figure 4:**
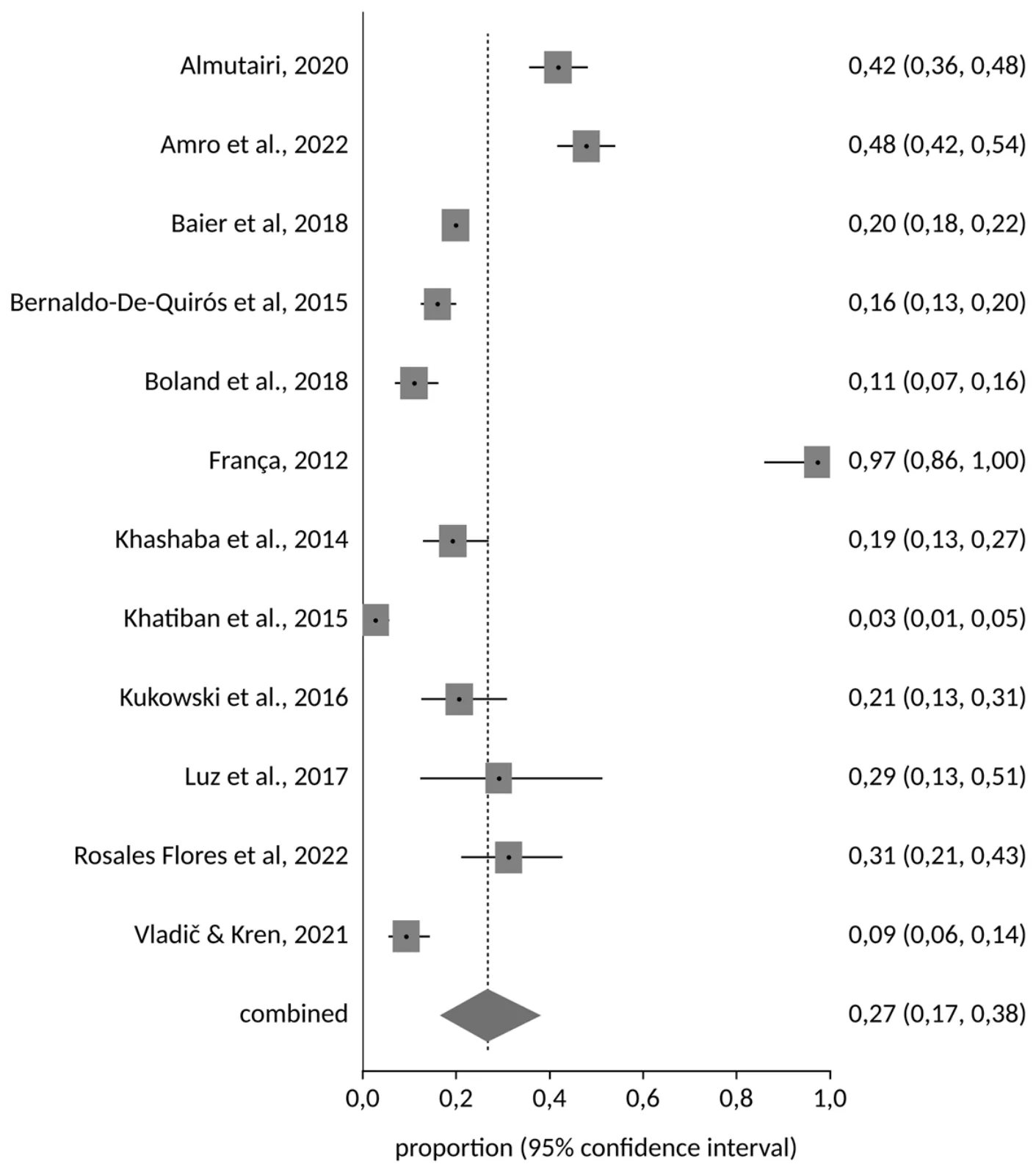
Forest plot of the pooled prevalence of low personal accomplishment (random-effects model).

Twelve studies (n = 3130) contributed to each of the three meta-analyses. The pooled prevalence was 33% for high EE (95% CI 22–45%; 95% prediction interval [PI] 1–81%; I^2^ = 97.7%) (Figure 2), 37% for high D (95% CI 26–48%; PI 4–79%; I^2^ = 97.1%) (Figure 3) and 27% for low PA (95% CI 17–38%; PI 0.3–72%; I^2^ = 97.4%) (Figure 4). Heterogeneity was very high, and the confidence intervals of the three dimensions overlapped. Egger’s test showed no evidence of small-study effects for EE (*p* = 0.37), D (*p* = 0.71), or low PA (*p* = 0.39), although its power is limited with twelve studies. According to GRADE, the certainty of the evidence was very low for all three estimates, downgraded for risk of bias (only 5 of the 12 pooled studies met at least seven JBI criteria), inconsistency (I^2^ ≥ 97% and wide prediction intervals), and imprecision (95% CIs spanning more than 20 percentage points) (Table 2).

Among the pooled studies, the prevalence of high D exceeded that of high EE in seven [17,48,50,51,54,55,56], whereas EE predominated in five [49,53,57,58,59].

### 3.5. Subgroups and Sensitivity Analyses

The results of the sensitivity and subgroup analyses are shown in Table 2. Leave-one-out estimates ranged from 28.5% to 36.4% for EE, from 30.2% to 39.7% for D, and from 20.8% to 29.8% for low PA. França et al. [56] (n = 38 nurses), which reported extreme values (89% high EE, 100% high D, and 97% low PA) using non-standard cut-offs, was the most influential study. It was retained in the main analysis because its values were confirmed against the original article; omitting it yielded the lowest leave-one-out estimates for the three dimensions (28.5%, 30.2% and 20.8%, respectively), and heterogeneity remained high (I^2^ 96.2–97.5%). Excluding the two studies that included non-eligible staff not reported separately [50,51] yielded 37.9%, 40.3% and 28.2%, respectively. No significant differences were found by region (EE 20.9% in Europe vs. 40.5% in other regions, *p* = 0.078; D *p* = 0.28; low PA *p* = 0.47). Only two pooled studies collected data during the COVID-19 pandemic [50,53]; their prevalences (EE 23% and 35%; D 28% and 32%; low PA 31% and 48%, respectively) fell within the range of the studies conducted before the pandemic (pooled EE 34%, D 38%, and low PA 24%) and are presented descriptively. In Khatiban et al. [58], whose low PA category corresponds to a PA score ≤ 33 in the original article, the prevalence of low PA was the lowest (2.7%); omitting this study increased the pooled estimate to 29.8%.

Subgroup analyses by professional role, instrument version, or cut-off were not feasible because few pooled studies reported these data separately.

### 3.6. Factors Associated with Burnout

#### 3.6.1. Sociodemographic Factors

Findings on gender were mixed. Women showed higher stress, EE, or overall burnout in several studies [36,41,45,53,54], whereas men showed higher burnout in two studies [22,50] and lower PA in two [19,54], and another study found no differences by sex [37]. Regarding age, younger workers reported higher burnout in several studies [38,53] and in the whole sample of [43], which also included hospital staff; in that whole sample, workers over 50 years had a lower risk than those aged 18–30 [43], whereas in another study the highest EE was found in those aged 40–49 years [58]. Younger professionals were also the most exposed to verbal and physical violence [17], and professionals with children had higher EE [37].

#### 3.6.2. Professional Role and Experience

Findings on professional role were mixed. Physicians were more affected than other professionals in three studies [43,49,54]; however, ref. [43] compared roles only in the whole sample, 92% of whom also worked in hospital emergency departments, and ref. [49] included only eight physicians, whereas two studies found no differences between professional categories [36,37]. Technicians showed higher EE in one study [50], and in a UK study, paramedics had the highest prevalence of personal and work-related burnout, technicians the highest prevalence of patient-related burnout, and emergency care assistants the lowest prevalence in all domains [22]. The only study conducted exclusively in nurses reported burnout in 76.3% of a small sample (n = 38) [56]. Findings on professional experience were also inconsistent: shorter experience was associated with higher burnout in one study [50] and in the whole sample of [43], whereas longer experience was associated with higher burnout or EE in others [22,54,58], with a non-significant trend in the same direction in another [37].

#### 3.6.3. Workplace Violence

Exposure to violence was frequent, mainly verbal: 86.5% of ambulance staff in one study [18] and 85.5% in another [40] had experienced verbal violence. Burnout was associated with exposure to violence in several studies [17,18,19,40]; in one of them, verbal, physical and sexual violence were associated with higher EE and D, and verbal violence with lower PA [40]. Women were more exposed to psychological violence in one study [18], whereas men experienced more physical threats in another [19]. Post-traumatic stress and workplace violence were also positively associated with burnout among paramedics [35].

#### 3.6.4. Organizational Factors

Burnout was associated with organizational factors [56], low job satisfaction [36] and lower quality of work life [33], although the latter study reported inconsistent correlation coefficients (r = −0.553 in the abstract and −0.624 in its results table). Lack of social support from supervisors and colleagues and poor communication predicted exhaustion, cynicism and lower competence [46], and low supervisor support, high work–family conflict and full-time work were independently associated with burnout [44]. Lack of time, frequent shifts of 24 h or longer and the absence of space to eat, exercise or rest [21], as well as lack of compensation for COVID-19-related overtime [45], were also associated with burnout. Working more than 60 h per week [49] and longer shifts [58] were associated with higher burnout, and poor sleep quality correlated with all three dimensions [54]. In a mixed-methods study, ambulance staff attributed burnout to four themes: external factors (mainly call-outs to patients who did not require an ambulance), patterns of work, management support and sickness absence [22]. Exposure to critical incidents, especially those involving children, was among the most difficult experiences reported [51,59].

## 4. Discussion

### 4.1. Main Findings

This review of 31 studies including 9926 prehospital professionals found that approximately one in three professionals had high EE (33%), high D (37%), or low PA (27%). The confidence intervals of the three dimensions overlapped widely, so no dimension can be considered clearly more affected than the others, and omitting one small study with extreme values [56] reduced the three estimates to 21–30%.

### 4.2. Comparison with Previous Reviews

These estimates are consistent with previous syntheses in prehospital settings. A recent meta-analysis of emergency medical services personnel reported an overall burnout prevalence of 40% (95% CI 33–49%; prediction interval 9–82%) [30], and a systematic review of paramedics found prevalences between 16% and 56%, which could not be pooled because of the heterogeneity of instruments [28]. Our review adds dimension-specific pooled estimates based on the MBI and a synthesis of associated factors restricted to prehospital professionals.

The very high heterogeneity (I^2^ 97–98%) and the wide prediction intervals indicate that the true prevalence differs substantially between services: in a new, comparable setting, the proportion of professionals with high EE could plausibly range from about 1% to 81% (95% prediction interval; Section 3.4). The pooled estimates should therefore be interpreted as an average across heterogeneous settings rather than as a single prevalence. This heterogeneity probably reflects (i) methodological differences, such as MBI versions and normative, country-specific or sample-based cut-offs; (ii) the professional composition of the samples, from technicians only to mixed teams or nurses only [56]; (iii) differences between emergency medical services systems (physician-staffed versus paramedic-based models, public versus private providers and shift length); and (iv) period effects, such as the COVID-19 pandemic. Region did not explain this heterogeneity, although EE tended to be less frequent in European studies (21% vs. 41%; *p* = 0.078), and the two studies conducted during the pandemic did not differ clearly from the others.

### 4.3. Dimension-Specific Findings

Among the studies reporting both EE and D, high D more often exceeded high EE. In Maslach’s model, EE is usually the first manifestation and arises when work demands exceed personal resources, whereas D develops as a defensive distancing that mitigates this strain [4]. In prehospital work, repeated exposure to critical incidents, suffering and aggression may favor such distancing, which could explain the relatively high prevalence of D. Low PA, in contrast, may depend more on internal factors, such as job satisfaction [60,61], and on external factors, such as social recognition of the profession, professional rewards and perceived support [62].

### 4.4. Individual Factors: Gender, Age and Experience

Regarding gender, the findings of the included studies were mixed: some reported higher stress, EE, or overall burnout among women [36,41,45,53,54], others reported higher burnout or lower PA among men [19,22,50,54], and another found no differences [37]. In other healthcare settings, female gender has been proposed as a risk factor for burnout [61,63,64], and being a woman, younger age, and concern about patients’ progress have been described as predisposing factors [65,66]. The inconsistency observed in prehospital settings may reflect the predominantly male composition of the workforce, the different distribution of roles between men and women, and the small number of women in some samples, which limits statistical power.

Working in predominantly male ambulance services does not appear to be a risk factor for burnout among women [67]. However, women may experience professional isolation, and their sense of community or belonging to a team can be undermined by gender-based microaggressions from patients, colleagues, and staff, which may affect PA [68,69].

Findings on age were also mixed: younger workers reported higher burnout in several included studies, whereas in another the highest EE was found among those aged 40–49 years [58]. In other settings, lack of experience, greater empathy and longer working hours [70], lack of support from colleagues [23], and sensation seeking [23,71] have been proposed to explain higher burnout among younger professionals. The role of family responsibilities is also unclear: in one included study, professionals with children had higher EE [37], whereas other authors have suggested that having children may provide resources for coping with stress [70]. Findings on years of experience were likewise inconsistent, with longer experience associated with higher burnout in some studies [22,54,58] and shorter experience in others [43,50]. Because age and experience are strongly correlated, both patterns are plausible: cumulative exposure to critical incidents may increase burnout over time, whereas a healthy worker effect, whereby professionals with burnout leave prehospital services, may make older and more experienced workers appear less affected in cross-sectional samples.

### 4.5. Professional Role

The prevalence of burnout among physicians varies widely across studies, from 12% to 72% [72,73,74,75], and physicians have shown greater resilience than the general working population, with resilience inversely related to burnout [75]. In our review, the evidence on professional role was mixed: physicians were more affected in three studies [43,49,54], but one of them compared roles only in a sample that largely also worked in hospital emergency departments [43] and another included only eight physicians [49], and two studies found no differences between professional categories [36,37]. If confirmed, this pattern could reflect the greater clinical responsibility and decision-making burden of physicians at the scene. The only study conducted exclusively among prehospital nurses reported burnout in 76.3% of 38 nurses [56]; given its small size and extreme values, this figure cannot be generalized to prehospital nursing, and in other settings the prevalence of burnout among nurses ranges from 12% to 51% [76,77]. Another study suggested that nurses working in ambulance services experience less severe burnout than nurses in other services [78], and nurses have reported greater personal fulfillment than physicians [78,79]. Technicians and paramedics also showed high levels of burnout [22,50], and among technicians, burnout was associated with a fourfold higher intention to quit within one year [38], which is consistent with the literature describing burnout as more frequent among paramedics [80]. These differences may reflect differences in training, autonomy, exposure to the scene and the patient, and professional recognition.

### 4.6. Workplace Violence and Organizational Factors

Healthcare workers providing care outside hospitals face emotionally stressful situations, and exposure to physical and verbal violence emerged in the included studies as a factor associated with burnout. Violence was frequent, mainly verbal, affecting more than 85% of staff in two studies [18,40], and two-thirds of respondents had been assaulted by a patient at least once in another [51]. In other settings, clinical staff exposed to physical and non-physical workplace violence show higher rates of depression and anxiety [81] and higher EE and D scores [81,82]. Violent situations [19], aggression, and threats predispose workers to burnout [83,84], which in turn reduces the quality of care [84].

From an organizational perspective, a substantial body of the literature shows that working conditions strongly influence the development of burnout, and some authors even distinguish between job satisfaction and organizational satisfaction [16]. Lack of leader support has been associated with higher EE and D scores and lower job satisfaction [19,67]. Similarly, leaders who involve their teams, keep them informed, guide them, and recognize their contributions promote job satisfaction [85]. These findings are consistent with our results. Poor organizational communication, excessive bureaucracy, administrative workload, and the digitization of administrative tasks have also been related to high stress reactivity [70,86,87].

The nature of the calls received can also influence the work environment. Although self-harm and suicide are the most common incidents attended by out-of-hospital services, traffic accidents and medical emergencies, as well as attending seriously injured friends, acquaintances, or children, were ranked as the most severe stressors [16]. Other authors emphasize the impact of individual incidents over their accumulation, as a single highly distressing event may affect workers more than repeated exposure to incidents without emotional impact [51]. In a nationwide study of Norwegian ambulance personnel, working overtime was associated with higher severity of all ambulance-specific stressors [23]. Consistent with the role of call type, Miller [22] found that ambulance staff frequently attributed exhaustion to being dispatched to calls that were not genuine medical emergencies, which they described as a significant source of frustration and reduced well-being. In the included studies, frequent shifts of 24 h or longer [21], poor sleep quality [54] and working more than 60 h per week [49] were associated with burnout, and in another study, reducing shifts from 24 to 12 h was associated with improvements in overall attitude and a temporary decrease in burnout scores [88].

Other authors have described a greater predisposition to burnout among medical staff working in large cities, related to low income relative to high living costs, heavy traffic and greater competition in the sector [89], whereas working in districts with fewer than 20,000 inhabitants was associated with lower frequencies of all burnout dimensions [80].

These factors are probably interrelated rather than independent. The effect of violence on EE and D appeared greater when institutional support was lacking [17,19], and supervisor support, respect and recognition emerged as protective resources in different countries [38,44,46]. From a theoretical perspective, this pattern is consistent with the job demands–resources approach used in some included studies [21] and with Maslach and Leiter’s areas of worklife [4]: prehospital-specific demands (violence, critical incidents, long or irregular shifts and non-urgent calls) appear to be mainly related to EE and D, whereas the lack of resources (support, reward and community) may contribute to low PA. This suggests that burnout in prehospital professionals should be understood as a consequence of the fit between the person and the organization rather than as an individual vulnerability.

### 4.7. Strengths and Limitations

This review has several strengths, including a protocol registered in PROSPERO, a search of five databases including LILACS, the inclusion of Spanish- and Portuguese-language studies, sensitivity and subgroup analyses with prediction intervals, and a GRADE assessment of certainty. However, it also has limitations. First, almost all studies were cross-sectional, which precludes causal inference; the associated factors should therefore be interpreted as correlates. Second, heterogeneity was very high (I^2^ 97–98%) and the prediction intervals were wide, so the pooled estimates should be interpreted with caution; accordingly, the certainty of the evidence was rated as very low. Third, the studies used different MBI versions and cut-offs, and three used the CBI, which measures a different construct; this limits comparability and prevented both the pooling of CBI studies and subgroup analyses by instrument or cut-off. Fourth, only 12 studies contributed to each meta-analysis, which limits the power of Egger’s test and of the subgroup analyses, whose results are exploratory. Fifth, several studies were conducted during the COVID-19 pandemic, which may have increased burnout levels, and only two pooled studies collected data during the pandemic, which precluded a formal comparison. Sixth, the search was restricted to English, Spanish, and Portuguese, did not include the grey literature, and required instrument terms in the search string, which may have missed eligible studies, a risk only partly mitigated by citation searching. In addition, three studies included small proportions of staff not covered by our eligibility criteria (ambulance drivers, volunteers, or dispatchers) without reporting them separately [36,50,51], and excluding the two pooled studies affected [50,51] did not substantially change the estimates. In another study, most participants also worked in hospital emergency departments; only its separately reported prehospital prevalence was extracted, and its associated factors refer to the whole sample [43].

A limitation of the included literature is the presence of small studies, such as that of França et al. [56], which yielded extreme estimates (e.g., 100% high D) that can distort pooled proportions and contribute to heterogeneity.

Finally, most studies used non-probabilistic sampling and self-reported measures, with a risk of selection and response bias. Future research should prioritize longitudinal designs, probabilistic sampling, standardized reporting of the MBI version, cut-offs and dimension-specific prevalences, stratification by professional role, and multicenter studies with comparable samples in different countries.

### 4.8. Implications for Clinical Practice and Research

The results of this review reinforce the need to rethink the approach to burnout interventions. Beyond individual-centered interventions, addressing institutional dynamics offers an opportunity to transform the work environment of healthcare professionals [56]. The priority should be to strengthen the resources available to these professionals and to foster organizational structures that protect their mental health.

Preventive interventions are essential, such as analyzing workload, rethinking working hours, and changing the organizational culture to incorporate values that protect against burnout and promote the emotional well-being of professionals [15]. Key elements will be fostering peer support and participation in decision-making, improving communication with management teams, and strengthening professional autonomy [29].

Psychosocial support strategies, such as debriefing groups or accessible psychological support units tailored to professionals, are also recommended [90]. In addition, continuing education should include content on self-care and stress management, providing professionals with personal tools to cope with emotionally charged situations [91].

## 5. Conclusions

In out-of-hospital emergency healthcare professionals, the pooled prevalence of high emotional exhaustion, high depersonalization and low personal accomplishment was 33%, 37% and 27%, respectively, with no clear predominance of any dimension. Heterogeneity between studies was very high, and the certainty of these estimates was very low, so they should be interpreted with caution. The most consistent associated factors were exposure to workplace violence, lack of organizational and supervisor support, and demanding shift patterns, whereas the evidence for professional role, gender, age, and years of experience was inconsistent. Prehospital services should incorporate periodic burnout screening, violence-prevention protocols, supervisory support, and shift schedules that ensure adequate rest. Future studies should use standardized instruments and cut-offs and longitudinal designs.

## Figures and Tables

**Table 1:** Characteristics of the included studies (ordered by reference number).

Ref.	Study (Country; Design)	Sample	Instrument (Version; Cut-Off)	Overall Burnout	EE	D	PA	Associated Factors	JBI
[17]	Bernaldo-De-Quirós et al., 2015 (Spain, Madrid; cross-sectional)	N = 441 (437 with burnout data); physicians, nurses, EMT	MBI-HSS (Spanish adaptation, Seisdedos; cut-offs for Spanish physicians)	–	High 9.2% (40/437) *	High 13.3% (58/437) *	Low PA 16.0% (70/437) *	Exposure to verbal and physical aggression associated with higher EE and D; lack of institutional support	9
[18]	Coskun Cenk, 2019 (Turkey, Muğla; cross-sectional, web survey April–June 2017)	N = 143 ambulance staff; 58.7% women; 57.3% paramedics	MBI (Turkish adaptation, Ergin)	–	12.07 (6.57)	7.97 (3.82)	9.16 (5.14)	86.5% exposed to verbal abuse and 35% to physical violence; MBI scores differed by sex, age, profession, and exposure to verbal abuse; women more exposed to psychological violence	5
[19]	Deniz et al., 2016 (Turkey, Kırıkkale; cross-sectional, 2013)	N = 120; age 29.5 (6.5); ambulance staff	MBI (22 items; 5-point scale, 0–4; mean scores)	–	11.59 (6.20)	4.34 (3.01)	9.16 (4.24)	Exposure to violence and lack of institutional support associated with higher EE and D; men: more physical threats and lower PA	4
[21]	Crowe et al., 2020 (USA, South Carolina; cross-sectional)	N = 1276; age 37.5 (9.8); EMS professionals	CBI work-related burnout subscale (CBI guidelines)	35%	–	–	–	Lack of time, frequent ≥24-h shifts, absence of space to eat, exercise or rest; supportive work environment protective	9
[22]	Miller, 2026 (UK, Yorkshire; mixed-methods survey, June 2019)	N = 78 (47 paramedics, 15 technicians, 16 emergency care assistants); 57.7% men; response rate 67.8%	CBI (burnout ≥ 50)	Personal 48.7%; work-related 53.8%; patient-related 37.1%	–	–	–	Highest risk: full-time men with >10 years of experience working as paramedics; paramedics highest in personal and work-related burnout, technicians in patient-related burnout. Themes: external factors (non-urgent call-outs), patterns of work, management support, sickness absence	6
[33]	Nabi-Amjad et al., 2023 (Iran, Chaharmahal and Bakhtiari; descriptive-correlational, data 2021)	N = 295; prehospital emergency staff (69.6% emergency technicians); age 34.2 (7.8)	MBI (22 items; total score 0–132)	Mean 81.1 (53.4)	–	–	–	Inverse correlation between quality of work life and burnout (r = −0.553 in the abstract; −0.624 in the results table)	6
[34]	Thielmann et al., 2024 (Germany; cross-sectional online survey)	N = 285; age 37.6 (10.4); EMS personnel	MBI-GS (German version, 16 items; mean item score 0–6)	–	Exhaustion: mean 2.1–3.3 across resilience levels	Cynicism: mean 1.7–2.5	Professional efficacy: mean 4.4–5.3	39% had low resilience; higher resilience associated with lower exhaustion, cynicism, and irritation	5
[35]	Sung & Lee, 2025 (South Korea, Busan; cross-sectional)	N = 208; fire-department paramedics; age 37.5 (7.1)	MBI (Korean version, 22 items; mean item score 0–6)	Mean 2.40 (1.07)	–	–	–	Post-traumatic stress and workplace violence positively, and resilience negatively, correlated with burnout (path analysis)	6
[36]	Iorga et al., 2015 (Romania; cross-sectional)	N = 122; physicians, nurses, and ambulance drivers (not reported separately)	MBI (total score)	Not reported numerically	–	–	–	Female sex, low job satisfaction, and alexithymia associated with higher burnout; no differences between professional categories	1
[37]	Sánchez-Rodríguez et al., 2001 (Spain, Madrid SAMUR; cross-sectional)	N = 73 (23 physicians, 12 nurses, 37 ambulance technicians); 79.5% men	MBI (Spanish validated version)	Mean 38.8 (9.5)	13.6 (4.5)	7.9 (2.6)	16.9 (5.0)	Professionals with children had higher EE; longer seniority was associated with higher EE and burnout (n.s.); no differences by sex or professional category	5
[38]	Koval et al., 2020 (India, four states; cross-sectional, July–November 2017)	N = 296 EMT-Basics (of 327 eligible); 73% men; median age 30	MBI-Medical Personnel Survey (local languages); burnout = EE ≥ 27 or D ≥ 10	28.7%	Median 18 (IQR 12–26)	Median 5 (IQR 2–8)	Median 42 (IQR 37–47)	Younger age, perceived lack of respect from colleagues and administrators, and sense of physical risk; burnout associated with fourfold intention to quit within one year	8
[39]	Tunaligil et al., 2016 (Turkey, Istanbul; cross-sectional, 2011)	N = 842 EMT-Basics (606 public, 236 private)	MBI (Turkish version)	–	Subgroup mean 23.1–27.2 (SD 4.7–7.8)	Subgroup mean 11.9–14.2	Subgroup mean 55.0–62.5	No differences between public and private EMTs in EE, D, or PA (*p* > 0.6); formal training, false accusations, work-related injuries, work disability, and organizational support associated with burnout; private sector: commute time	7
[40]	Avcı & Demirci, 2026 (Turkey; cross-sectional)	N = 1013 (652 paramedics, 361 EMT)	MBI-HSS (Turkish version, 5-point)	–	18.3 (8.0)	7.0 (3.9)	10.5 (4.2)	Verbal, physical, and sexual violence associated with higher EE and D; verbal violence with lower PA	6
[41]	Wongtongkam, 2017 (Australia; cross-sectional)	N = 48; paramedics; age 39.2 (10.2)	Abbreviated MBI (12 items, McManus)	Total: men 39.5 (8.5); women 39.1 (5.8)	Men 5.30 (4.62); women 8.36 (3.51)	Men 4.40 (4.29); women 5.05 (3.67)	Men 14.10 (3.07); women 12.89 (2.92)	Women: significantly higher EE and lower career satisfaction	4
[42]	Abareshi et al., 2022 (Iran; cross-sectional)	N = 198; age 29.5 (5.4); EMS staff (80.3% medical emergency technicians)	MBI (22 items); sample-based tertile cut-offs (P33/P66)	Mean 51.4 (11.3)	13.9 (7.2); high 6.1% §	10.1 (3.6); high 32.8% §	26.6 (6.7); category direction unclear §	Mental workload (overall workload) associated with EE and D; educational level and occupational category associated with burnout	5
[43]	Liu et al., 2024 (China, Chengdu; cross-sectional, July–August 2023)	N = 181 full-time prehospital staff extracted (of 2299 participants; 92% also worked in hospital emergency departments)	MBI-GS (Chinese version, 15 items; total score)	Full-time prehospital staff: 38.1% (69/181). Whole sample: 33.4% (mild 30.27%; moderate 2.78%; severe 0.3%)	–	–	–	Whole sample (not reported separately): physicians more prone than nurses; 0–5 years of experience higher risk than 6–10 or >15 years; >50 years lower risk than 18–30; higher fatigue	6
[44]	Haruna et al., 2023 (Japan; cross-sectional, during COVID-19)	N = 700 EMTs (673 after excluding missing data)	MBI-HSS; burnout = EE ≥ 27 and/or D ≥ 10	25.6%	–	–	–	Low supervisor support (OR 1.42, 95% CI 1.29–1.57), high family–work negative spillover (OR 1.26, 1.14–1.41) and full-time work (OR 2.04, 1.23–3.38)	7
[45]	Kim et al., 2023 (Korea, Seoul; cross-sectional online survey, June 2021)	N = 693 EMS providers; 82.5% men	CBI (Korean version; personal, work-related, and client-related burnout scores)	Domain mean values not reported in the main text	–	–	–	Lack of compensation for COVID-19-related overtime associated with higher burnout in all domains; higher burnout scores in women	7
[46]	van der Ploeg & Kleber, 2003 (Netherlands; longitudinal, 1-year follow-up)	N = 221 at baseline (123 at follow-up); ambulance workers; 86% men	MBI-GS (Dutch version, 15 items); Schaufeli & van Dierendonck criteria	8.6% at risk of burnout	High exhaustion 12%	High cynicism 18%	Low competence 16%	Lack of social support from supervisors and colleagues and poor communication predicted exhaustion, cynicism, and lower competence	6
[47]	Roth et al., 2025 (Germany; cross-sectional)	N = 1024 EMS workers (EMSiG survey, 2015; same project as [48]); nurses not extracted	MBI-HSS; EE ≥ 27, D ≥ 10 (3rd ed. manual)	–	High 25.8%; moderate 30.5%; low 43.8%	High 39.6%; moderate 34.8%; low 25.7%	Not assessed	Workplace violence associated with EE and D. Overlapping sample with [48]: not pooled; narrative synthesis only	6
[48]	Baier et al., 2018 (Germany; cross-sectional, EMSiG online survey June–December 2015)	N = 1101; EMS workers	MBI-HSS; EE ≥ 27, D ≥ 13, PA ≤ 31	–	High 25.3%	High 40.2%	Low PA 19.9%	–	6
[49]	Luz et al., 2017 (Brazil; cross-sectional)	N = 32 (24 healthcare professionals extracted: 8 physicians, 8 nurses, 8 nursing technicians/assistants; 8 drivers excluded)	MBI-HSS; Lautert cut-offs (P75/P25)	–	High 37.5% (9/24)	High 29.2% (7/24)	Low PA 29.2% (7/24) ‡	Physicians: highest EE (87.5%) and D (75%); >60 h/week associated with higher EE	7
[50]	Rosales-Flores et al., 2022 (Mexico; cross-sectional, 2020 pandemic)	N = 80; prehospital personnel (71.3% technicians; 25% volunteers, not reported separately)	MBI-HSS (Spanish version)	–	High 22.5%; moderate 17.5%; low 60%	High 27.5%; moderate 23.5%; low 49%	Low PA 31.25%; moderate 37.5%; high PA 31.25%	Male sex, technician role, shorter tenure, public institution, and full-time contract associated with higher EE	4
[51]	Boland et al., 2018 (USA, Minnesota; cohort, cross-sectional analysis)	N = 209 (190 paramedics/EMT, 19 dispatchers); age 40 (12)	MBI-HSS; US normative cut-offs (EE ≥ 27, D ≥ 13, PA ≤ 31)	18%	High 6%; moderate 22%; low 72%	High 15%; moderate 29%; low 56%	Low PA 11% ‡; moderate 33%; high PA 56%	Exposure to critical incidents, especially involving children	6
[53]	Amro et al., 2022 (Spain, Barcelona; cross-sectional, March–April 2021)	N = 280; age 41; EMT	MBI-HSS; EE ≥ 30, D ≥ 12, PA ≤ 33	–	High 35%; moderate 37%; low 28%	High 32%; moderate 40%; low 28%	Low PA 48%; moderate 30%; high PA 22%	Female sex, age 21–40, lack of PPE and fear of infection associated with stress; stress correlated with EE and D	6
[54]	Vladič & Kren, 2021 (Slovenia; cross-sectional, February–May 2019)	N = 204; prehospital EMS employees	MBI-HSS (Slovenian version)	–	High 17.6%; moderate 79.9%; low 2.5%	High 32.8%; moderate 63.7%; low 3.4%	Low PA 9.3%; moderate 78%; high PA 12.8%	Poor sleep quality correlated with EE, D, and PA; women had higher EE and D; physicians were most affected in EE and D; >10 years of service increased EE	5
[55]	Kukowski et al., 2016 (Canada; cross-sectional)	N = 87; age 42.1; paramedics	MBI-HSS; EE ≥ 27, D ≥ 10, PA ≤ 33	–	High 49%	High 67%	Low PA 21%	Higher PA buffered the effect of stress on sleep	7
[56]	França et al., 2012 (Brazil, Alagoas; cross-sectional, September 2010)	N = 38; nurses	MBI; cut-offs of the Center for Advanced Studies on Burnout Syndrome (EE ≥ 26, D ≥ 9, PA ≤ 33)	76.3%	High 88.9% †	High 100%	Low PA 97.4%	Burnout was more related to organizational factors than to the type of activity	6
[57]	Almutairi & El Mahalli, 2020 (Saudi Arabia, Riyadh; cross-sectional)	N = 270; EMS	MBI-HSS; scoring-key cut-offs	–	High 63.0%; moderate 25.9%; low 11.1%	High 40%; moderate 29.6%; low 30.4%	Low PA 41.9%; moderate 36.7%; high PA 21.5%	Nationality and coping methods associated with EE, D and PA	7
[58]	Khatiban et al., 2015 (Iran, Kermanshah and Hamadan; cross-sectional)	N = 260; age 40; EMS	MBI (Persian version; frequency and intensity); frequency cut-offs EE ≥ 30, D ≥ 12, PA ≤ 33	–	High frequency 46.5%	High frequency 38.8%	Low PA (high frequency of lack of PA) 2.7% ‡	Age 40–49, single status, smoking, lower income, longer experience, and longer shifts associated with higher burnout	7
[59]	Khashaba et al., 2014 (Egypt, Mansoura; comparative cross-sectional)	N = 140 EMR (vs. 140 comparison group); age 31.6 (6.4)	MBI; EE > 30, D > 12, PA < 33	–	High 20%; low/moderate 80%	High 9.3%; low/moderate 90.7%	Low PA (high burnout, <33) 19.3% ‡; low/moderate burnout 80.7%	Most severe acute stressors: traumatic events, severe accidents, young victims	6

§ In [42], categories were defined by sample tertiles with overlapping score ranges; not pooled. * In [17], recalculated as the weighted total of the three violence-exposure groups (Table 3 of the original article). † Reported percentage; not consistent with n = 38 (closest 34/38). Values are percentages of participants in each category or mean (SD) scores unless otherwise stated. EE and D categories refer to the level of the score (high = burnout-indicative). PA categories refer to the personal accomplishment score; low PA indicates burnout. ‡ PA categories were converted from burnout levels or inverse scales in four studies: [51] (the categories of the original table are labelled in the burnout direction; the 11% corresponds to the category reported as high, which is consistent with the reported mean PA of 39.1, SD 6.2, and was not estimated from the mean), [59] (high burnout on the PA subscale), [58] (high frequency of lack of PA, i.e., PA ≤ 33) and [49] (professional incompetence). For [53], the low-PA value (48%) is taken from the text of the original article; the 22% given in the first submission corresponded to high PA. CBI, Copenhagen Burnout Inventory; D, depersonalization; EE, emotional exhaustion; EMS, emergency medical services; EMT, emergency medical technician; IQR, interquartile range; MBI, Maslach Burnout Inventory; JBI, Joanna Briggs Institute checklist score (number of items met out of 9); MBI-GS, Maslach Burnout Inventory–General Survey; MBI-HSS, Maslach Burnout Inventory–Human Services Survey; OR, odds ratio; PA, personal accomplishment; PPE, personal protective equipment.

**Table 2:** Sensitivity and subgroup analyses of the pooled prevalence of burnout dimensions.

Analysis	High EE % (95% CI); k	High D % (95% CI); k	Low PA % (95% CI); k
Main analysis	33.2 (22.1–45.3); 12	36.7 (26.3–47.8); 12	26.7 (17.0–37.7); 12
95% prediction interval	1.0–80.5	3.9–79.2	0.3–71.9
I^2^; τ^2^	97.7%; 0.0451	97.1%; 0.0360	97.4%; 0.0398
Leave-one-out range	28.5–36.4	30.2–39.7	20.8–29.8
Omitting França [56]	28.5 (18.3–39.9); 11	30.2 (21.7–39.5); 11	20.8 (13.1–29.8); 11
Excluding studies with non-eligible staff [50,51]	37.9 (25.4–51.1); 10	40.3 (28.5–52.6); 10	28.2 (17.0–41.0); 10
Region: Europe	20.9 (11.4–32.5); 4	29.0 (16.2–43.6); 4	21.9 (10.8–35.7); 4
Region: other regions	40.5 (21.9–60.7); 8	41.5 (23.9–60.2); 8	29.9 (13.4–49.6); 8
*p* (Q-between)	0.078	0.28	0.47
Data collected before COVID-19 (pooled)	34.2 (21.1–48.6); 10	38.3 (25.9–51.5); 10	24.2 (14.6–35.2); 10
Data collected during COVID-19 [50,53] (individual studies)	22.5; 35.0	27.5; 32.1	31.3; 47.9
Egger test, *p*	0.37	0.71	0.39
Certainty of evidence (GRADE)	Very low ^a^	Very low ^a^	Very low ^a^

Random-effects models (Freeman–Tukey double arcsine transformation, DerSimonian–Laird estimator); τ^2^ on the transformed scale. Studies conducted during the COVID-19 pandemic are shown individually because a pooled comparison of two studies is not interpretable. ^a^ Downgraded for risk of bias (5 of 12 studies met ≥7 JBI criteria), inconsistency (I^2^ ≥ 97%; wide prediction intervals), and imprecision (95% CI width > 20 percentage points). GRADE, Grading of Recommendations Assessment, Development and Evaluation; JBI, Joanna Briggs Institute; EE, emotional exhaustion; D, depersonalization; PA, personal accomplishment; CI, confidence interval; k, number of studies.

## Data Availability

The data presented in this study are available on request from the corresponding author due to ethical reasons.

## Supplementary Materials

The following supporting information can be downloaded at https://www.mdpi.com/article/10.3390/healthcare14213591/s1. Table S1: PRISMA 2020 checklist; Table S2: Complete search strategies for each database; Table S3: Risk of bias of the included studies assessed with the JBI Critical Appraisal Checklist for Studies Reporting Prevalence Data. References [17,18,19,21,22,33,34,35,36,37,38,39,40,41,42,43,44,45,46,47,48,49,50,51,53,54,55,56,57,58,59] are cited in the Supplementary Materials.

## References

[1] Burn-Out an “Occupational Phenomenon”: International Classification of Diseases. https://www.who.int/news/item/28-05-2019-burn-out-an-occupational-phenomenon-international-classification-of-diseases.

[2] World Health Organization (2026). ICD-11 for Mortality and Morbidity Statistics [Internet].

[3] Maslach C., Schaufeli W.B., Leiter M.P. (2001). Job burnout. Annu. Rev. Psychol..

[4] Maslach C., Schaufeli W.B., Maslach C., Marek T. (1993). Burnout: A Multidimensional Perspective. Professional Burnout: Recent Developments in Theory and Research.

[5] Güler Y., Şengül S., Çaliş H., Karabulut Z. (2019). Burnout syndrome should not be underestimated. Rev. Assoc. Med. Bras..

[6] Sullivan D., Sullivan V., Weatherspoon D., Frazer C. (2022). Comparison of Nurse Burnout, Before and During the COVID-19 Pandemic. Nurs. Clin. N. Am..

[7] Denning M., Goh E.T., Tan B., Kanneganti A., Almonte M., Scott A., Martin G., Clarke J., Sounderajah V., Markar S. (2021). Determinants of burnout and other aspects of psychological well-being in healthcare workers during the COVID-19 pandemic: A multinational cross-sectional study. PLoS ONE.

[8] Zhang X., Wang J., Hao Y., Wu K., Jiao M., Liang L., Gao L., Ning N., Kang Z., Shan L. (2021). Prevalence and Factors Associated With Burnout of Frontline Healthcare Workers in Fighting Against the COVID-19 Pandemic: Evidence From China. Front. Psychol..

[9] Williams E.S., Manwell L.B., Konrad T.R., Linzer M. (2007). The relationship of organizational culture, stress, satisfaction, and burnout with physician-reported error and suboptimal patient care: Results from the MEMO study. Health Care Manag. Rev..

[10] López-Cortacans G., Pérez-García M., Pérez-García R., Pérez-García D. (2021). Síndrome de burnout y efectos adversos: La prevención ineludible. Aten. Primaria.

[11] Han S., Shanafelt T.D., Sinsky C.A., Awad K.M., Dyrbye L.N., Fiscus L.C., Trockel M., Goh J. (2019). Estimating the Attributable Cost of Physician Burnout in the United States. Ann. Intern. Med..

[12] Dewa C.S., Loong D., Bonato S., Thanh N.X., Jacobs P. (2014). How does burnout affect physician productivity? A systematic literature review. BMC Health Serv. Res..

[13] Hamidi M.S., Bohman B., Sandborg C., Smith-Coggins R., de Vries P., Albert M.S., Murphy M.L., Welle D., Trockel M.T. (2018). Estimating institutional physician turnover attributable to self-reported burnout and associated financial burden: A case study. BMC Health Serv. Res..

[14] Hermoso de Mendoza Urrizola J. (2004). Absentismo asociado al síndrome de burnout. Coste económico del estrés laboral asistencial en un hospital. Mapfre Med..

[15] Lawn S., Roberts L., Willis E., Couzner L., Mohammadi L., Goble E. (2020). The effects of emergency medical service work on the psychological, physical, and social well-being of ambulance personnel: A systematic review of qualitative research. BMC Psychiatry.

[16] Alexander D.A., Klein S. (2001). Ambulance personnel and critical incidents: Impact of accident and emergency work on mental health and emotional well-being. Br. J. Psychiatry.

[17] Bernaldo-De-Quirós M., Piccini A.T., Gómez M.M., Cerdeira J.C. (2015). Psychological consequences of aggression in pre-hospital emergency care: Cross sectional survey. Int. J. Nurs. Stud..

[18] Cenk S.C. (2019). An analysis of the exposure to violence and burnout levels of ambulance staff. Turk. J. Emerg. Med..

[19] Deniz T., Saygun M., Eroğlu O., Ülger H., Azapoğlu B. (2016). Effect of exposure to violence on the development of burnout syndrome in ambulance staff. Turk. J. Med. Sci..

[20] Patterson P.D., Weaver M.D., Frank R.C., Warner C.W., Martin-Gill C., Guyette F.X., Fairbanks R.J., Hubble M.W., Songer T.J., Callaway C.W. (2012). Association between poor sleep, fatigue, and safety outcomes in emergency medical services providers. Prehosp. Emerg. Care.

[21] Crowe R.P., Fernandez A.R., Pepe P.E., Cash R.E., Rivard M.K., Wronski R., Anderson S.E., Hogan T.H., Andridge R.R., Panchal A.R. (2020). The association of job demands and resources with burnout among emergency medical services professionals. J. Am. Coll. Emerg. Physicians Open.

[22] Miller E. (2026). The prevalence of burnout in UK emergency ambulance staff: A mixed-methods survey study. Br. Paramed. J..

[23] Sterud T., Hem E., Ekeberg Ø., Lau B. (2008). Occupational stressors and its organizational and individual correlates: A nationwide study of Norwegian ambulance personnel. BMC Emerg. Med..

[24] Eisenberg R.L. (2022). Burnout. Acad. Radiol..

[25] Sullivan V., Hughes V., Wilson D.R. (2022). Nursing Burnout and Its Impact on Health. Nurs. Clin. N. Am..

[26] Bridgeman P.J., Bridgeman M.B., Barone J. (2018). Burnout syndrome among healthcare professionals. Am. J. Health Syst. Pharm..

[27] Batanda I. (2024). Prevalence of burnout among healthcare professionals: A survey at fort portal regional referral hospital. npj Ment. Health Res..

[28] Reardon M., Abrahams R., Thyer L., Simpson P. (2020). Review article: Prevalence of burnout in paramedics: A systematic review of prevalence studies. Emerg. Med. Australas..

[29] Thielmann B., Schwarze R., Böckelmann I. (2023). A Systematic Review of Associations and Predictors for Job Satisfaction and Work Engagement in Prehospital Emergency Medical Services—Challenges for the Future. Int. J. Environ. Res. Public Health.

[30] Huang G., Hsu C.W., Chou C.W., Hung W.K., Senevirathne B.J., Ngolombe R., Chisaka J.W., Chilinda Z.B., Banda K.J. (2026). Prevalence of burnout and associated moderator factors among emergency medical services personnel: A meta-analysis. Eur. J. Emerg. Med..

[31] Page M.J., McKenzie J.E., Bossuyt P.M., Boutron I., Hoffmann T.C., Mulrow C.D., Shamseer L., Tetzlaff J.M., Akl E.A., Brennan S.E. (2021). The PRISMA 2020 statement: An updated guideline for reporting systematic reviews. BMJ.

[32] Munn Z., Moola S., Lisy K., Riitano D., Tufanaru C. (2015). Methodological guidance for systematic reviews of observational epidemiological studies reporting prevalence and cumulative incidence data. Int. J. Evid.-Based Healthc..

[33] Nabi-Amjad R., Kaviyani S., Sadeghi-Gandomani H. (2023). The relationship between job burnout and quality of work life in pre-hospital emergency staff: Descriptive-correlation study. J. Multidiscip. Care.

[34] Thielmann B., Ifferth M., Böckelmann I. (2024). Resilience as Safety Culture in German Emergency Medical Services: Examining Irritation and Burnout. Healthcare.

[35] Sung J., Lee N. (2025). Post-Traumatic Stress, Workplace Violence, Resilience, and Burnout: A Path Analysis Among Korean Paramedics. Healthcare.

[36] Iorga M., Dascalu N., Soponaru C., Ioan B. (2015). Burnout syndrome among public ambulance staff. Rev. Med. Chir. Soc. Med. Nat. Iasi.

[37] Sánchez Rodríguez Manzaneque A., Lucas García N., García-Ochoa Blanco M.J., Sánchez Ferrer C., Jiménez Fraile J.A., Bustinza Arriortua A. (2001). Estrés laboral en el profesional de un servicio de emergencias prehospitalario. Emergencias.

[38] Koval K.W., Lindquist B., Gennosa C., Mahadevan A., Niknam K., Patil S., Rao G.V.R., Strehlow M.C., Newberry J.A. (2020). First look at emergency medical technician wellness in India: Application of the Maslach Burnout Inventory in an unstudied population. PLoS ONE.

[39] Tunaligil V., Dokucu A.I., Erdogan M.S. (2016). Determinants of General Health, Work-Related Strain, and Burnout in Public Versus Private Emergency Medical Technicians in Istanbul. Workplace Health Saf..

[40] Avcı K., Demirci Ş. (2026). Workplace violence and burnout in the prehospital emergency medical services: A cross-sectional study. Intern. Emerg. Med..

[41] Wongtongkam N. (2017). An exploration of violence against paramedics, burnout and post-traumatic symptoms in two Australian ambulance services. Int. J. Emerg. Serv..

[42] Abareshi F., Salimi F., Farnia F., Fallahi M., Rastaghi S. (2022). The impact of mental workload, work-related and socio-demographic factors on job burnout among emergency medical staff. Work.

[43] Liu Z., Luo L., Dai H., Zhang B., Ma L., Xiang T. (2024). An important issue of burnout among pre-hospital emergency medical personnel in Chengdu: A cross-sectional study. BMC Emerg. Med..

[44] Haruna J., Uemura S., Taguchi Y., Muranaka S., Niiyama S., Inamura H., Sawamoto K., Mizuno H., Narimatsu E. (2023). Influence of work and family environment on burnout among emergency medical technicians. Clin. Exp. Emerg. Med..

[45] Kim J.H., Yoon J., Kim S.J., Kim J.Y., Bahk J., Kim S.S. (2023). Lack of compensation for COVID-19-related overtime work and its association with burnout among EMS providers in Korea. Epidemiol. Health.

[46] van der Ploeg E., Kleber R.J. (2003). Acute and chronic job stressors among ambulance personnel: Predictors of health symptoms. Occup. Environ. Med..

[47] Roth K., Köppen J., Henschke C. (2025). Workplace violence and burnout among emergency medical service workers and nurses in Germany: A cross-sectional study. Hum. Resour. Health.

[48] Baier N., Roth K., Felgner S., Henschke C. (2018). Burnout and safety outcomes—A cross-sectional nationwide survey of EMS-workers in Germany. BMC Emerg. Med..

[49] Luz L.M., Barbosa Torres R.R., de Queiroga Sarmento K.M.V., Rocha Sales J.M., Nascimento Farias K., Braga Marques M. (2017). Burnout Syndrome in urgency mobile service professionals. Rev. Pesqui. Cuid. Fundam..

[50] Rosales Flores R.A., Durán Lizarraga M.E., López Ortiz C.A. (2022). Desgaste laboral en personal prehospitalario en México durante la pandemia de 2020. Salud Trab..

[51] Boland L.L., Kinzy T.G., Myers R.N., Fernstrom K.M., Kamrud J.W., Mink P.J., Stevens A.C. (2018). Burnout and Exposure to Critical Incidents in a Cohort of Emergency Medical Services Workers from Minnesota. West. J. Emerg. Med..

[52] Iorio A., Spencer F.A., Falavigna M., Alba C., Lang E., Burnand B., McGinn T., Hayden J., Williams K., Shea B. (2015). Use of GRADE for assessment of evidence about prognosis: Rating confidence in estimates of event rates in broad categories of patients. BMJ.

[53] Amro T.M., Arcos González P., Montero Viñuales E., Castro Delgado R. (2022). Impact of COVID-19 Pandemic on Stress and Burnout Levels amongst Emergency Medical Technicians: A Cross-Sectional Study in Spain. Ann. Med..

[54] Vladič M., Kren A. (2021). Sleep deprivation and burnout syndrome among pre-hospital emergency medical service employees in Slovenia: A cross-sectional study. Obz. Zdr. Nege.

[55] Kukowski C., King D.B., DeLongis A. (2016). Protective effect of paramedics’ sense of personal accomplishment at work: Mitigating the impact of stress on sleep. Australas. J. Paramed..

[56] França S.P., De Martino M.M.F., Aniceto E.V.S., Silva L.L. (2012). Predictors of Burnout Syndrome in nurses in the prehospital emergency services. Acta Paul. Enferm..

[57] ALmutairi M.N., El Mahalli A.A. (2020). Burnout and Coping Methods among Emergency Medical Services Professionals. J. Multidiscip. Healthc..

[58] Khatiban M., Hosseini S., Bikmoradi A., Roshanaei G., Karampourian A. (2015). Occupational Burnout and Its Determinants among Personnel of Emergency Medical Services in Iran. Acta Med. Iran..

[59] Khashaba E.O., Abdel Fattah El-Sherif M., Al-Wehedy Ibrahim A., Neatmatallah M.A. (2014). Work-Related Psychosocial Hazards Among Emergency Medical Responders (EMRs) in Mansoura City. Indian J. Community Med..

[60] Escribà-Agüir V., Artazcoz L., Pérez-Hoyos S. (2008). Efecto del ambiente psicosocial y de la satisfacción laboral en el síndrome de burnout en médicos especialistas. Gac. Sanit..

[61] Hacer T.Y., Ali A. (2020). Burnout in physicians who are exposed to workplace violence. J. Forensic Leg. Med..

[62] Maslach C., Leiter M.P. (2000). The Truth About Burnout: How Organizations Cause Personal Stress and What to Do About It.

[63] Raftopoulos V., Charalambous A., Talias M. (2012). The factors associated with the burnout syndrome and fatigue in Cypriot nurses: A census report. BMC Public Health.

[64] McMurray J.E., Linzer M., Konrad T.R., Douglas J., Shugerman R., Nelson K. (2000). The Work Lives of Women Physicians. J. Gen. Intern. Med..

[65] Dong M., Zhou F.C., Xu S.W., Zhang Q., Ng C.H., Ungvari G.S., Xiang Y. (2020). Prevalence of suicide-related behaviors among physicians: A systematic review and meta-analysis. Suicide Life Threat. Behav..

[66] Grau A., Flichtentrei D., Suñer R., Prats M., Braga F. (2009). Influencia de factores personales, profesionales y transnacionales en el síndrome de burnout en personal sanitario hispanoamericano y español (2007). Rev. Esp. Salud Pública.

[67] Sterud T., Hem E., Lau B., Ekeberg O. (2011). A comparison of general and ambulance specific stressors: Predictors of job satisfaction and health problems in a nationwide one-year follow-up study of Norwegian ambulance personnel. J. Occup. Med. Toxicol..

[68] Ahmad S.R., Ahmad T.R., Balasubramanian V., Facente S., Kin C., Girod S. (2022). Are You Really the Doctor? Physician Experiences with Gendered Microaggressions from Patients. J. Womens Health.

[69] Mete M., Dickman J., Rowe S., Trockel M.T., Rotenstein L., Khludenev G., Marchalik D. (2022). Beyond burnout: Understanding the well-being gender gap in general surgery by examining professional fulfillment and control over schedule. Am. J. Surg..

[70] Taranu S.M., Ilie A.C., Turcu A.M., Stefaniu R., Sandu I.A., Pislaru A.I., Alexa I.D., Sandu C.A., Rotaru T.-S., Alexa-Stratulat T. (2022). Factors Associated with Burnout in Healthcare Professionals. Int. J. Environ. Res. Public Health.

[71] Chng C.L., Eaddy S. (1999). Sensation seeking as it relates to burnout among emergency medical personnel: A Texas study. Prehosp. Disaster Med..

[72] Rotenstein L.S., Torre M., Ramos M.A., Rosales R.C., Guille C., Sen S., Mata D.A. (2018). Prevalence of Burnout Among Physicians: A Systematic Review. JAMA.

[73] Romani M., Ashkar K. (2014). Burnout among physicians. Libyan J. Med..

[74] Soler J.K., Yaman H., Esteva M., Dobbs F., Asenova R.S., Katic M., Ožvačić Z., Desgranges J.P., Moreau A., Lionis C. (2008). Burnout in European family doctors: The EGPRN study. Fam. Pract..

[75] West C.P., Dyrbye L.N., Sinsky C., Trockel M., Tutty M., Nedelec L., Carlasare L.E., Shanafelt T.D. (2020). Resilience and Burnout Among Physicians and the General US Working Population. JAMA Netw. Open.

[76] Nantsupawat A., Kunaviktikul W., Nantsupawat R., Wichaikhum O.A., Thienthong H., Poghosyan L. (2017). Effects of nurse work environment on job dissatisfaction, burnout, intention to leave. Int. Nurs. Rev..

[77] Palmer-Morales Y., Prince-Vélez R., Searcy-Bernal R., Compean-Saucedo B. (2007). Prevalence of burnout syndrome in nurses in 2 Mexican hospitals. Enferm. Clin..

[78] Popa F., Arafat R., Purcärea V., Lälä A., Bobîrnac G. (2010). Occupational Burnout levels in Emergency Medicine—A nationwide study and analysis. J. Med. Life.

[79] Sahraian A., Fazelzadeh A., Mehdizadeh A.R., Toobaee S.H. (2008). Burnout in hospital nurses: A comparison of internal, surgery, psychiatry and burns wards. Int. Nurs. Rev..

[80] Crowe R.P., Bower J.K., Cash R.E., Panchal A.R., Rodriguez S.A., Olivo-Marston S.E. (2018). Association of Burnout with Workforce-Reducing Factors among EMS Professionals. Prehosp. Emerg. Care.

[81] Shi L., Li G., Hao J., Wang W., Chen W., Liu S., Yu Z., Shi Y., Ma Y., Fan L. (2020). Psychological depletion in physicians and nurses exposed to workplace violence: A cross-sectional study using propensity score analysis. Int. J. Nurs. Stud..

[82] Molero Jurado M.M., Gázquez Linares J.J., Pérez-Fuentes M.C., Martos Martínez Á. (2023). Aggression and burnout in nurses during COVID-19: A cross-sectional study. Nurs. Health Sci..

[83] Erdur B., Ergin A., Yüksel A., Türkçuer İ., Ayrık C., Boz B. (2015). Assessment of the relation of violence and burnout among physicians working in the emergency departments in Turkey. Ulus. Travma Acil Cerrahi Derg..

[84] Arnetz J.E., Arnetz B.B. (2001). Violence towards health care staff and possible effects on the quality of patient care. Soc. Sci. Med..

[85] Shanafelt T.D., Gorringe G., Menaker R., Storz K.A., Reeves D., Buskirk S.J., Sloan J.A., Swensen S.J. (2015). Impact of Organizational Leadership on Physician Burnout and Satisfaction. Mayo Clin. Proc..

[86] Yates S.W. (2020). Physician Stress and Burnout. Am. J. Med..

[87] Gesner E., Dykes P.C., Zhang L., Gazarian P. (2022). Documentation Burden in Nursing and Its Role in Clinician Burnout Syndrome. Appl. Clin. Inform..

[88] Boudreaux E., Mandry C., Brantley P.J. (1998). Emergency Medical Technician Schedule Modification: Impact and Implications during Short- and Long-term Follow-up. Acad. Emerg. Med..

[89] Sporer C. (2021). Burnout in emergency medical technicians and paramedics in the USA. Int. J. Emerg. Serv..

[90] Huniche L., Milling L., Wittrock D., Mikkelsen S., Bruun H. (2024). Preventing burnout from moral distress among prehospital emergency personnel through action research and targeted clinical ethics support. Sci. Rep..

[91] Giaume L., Jimenez A., Kedzierewicz R., Jost D., Pery M., Trouvé J., Burlaton G., Travers S., Prunet B., Trousselard M. (2024). FIRECARE: An Evidence-Based Prevention Program to Reduce Burnout among Prehospital Caregivers: Benefits of a Combined Mindfulness, Heart Coherence Training, and Positive Psychology Intervention. Prehosp. Emerg. Care.

