# Supplementary material for: Burnout in Out-of-Hospital Emergency Healthcare Professionals: Systematic Review and Meta-Analysis
Source: Healthcare (Basel). 2026 Oct 23;14(21):3591. doi: 10.3390/healthcare14213591 (PMC13763490; doi:10.3390/healthcare14213591)
Supplement: Supplementary file 1 [file healthcare-14-03591-s001.zip › healthcare-4601080-supplementary.pdf]

Table S1. PRISMA 2020 checklist.

Section and Topic	Item #	Checklist item	Location where item is reported
**TITLE**			
Title	1	Identify the report as a systematic review.	Title
**ABSTRACT**			
Abstract	2	See the PRISMA 2020 for Abstracts checklist.	Abstract
**INTRODUCTION**			
Rationale	3	Describe the rationale for the review in the context of existing knowledge.	Section 1 (Introduction), paragraphs 1–7
Objectives	4	Provide an explicit statement of the objective(s) or question(s) the review addresses.	Section 1 (Introduction), last paragraph
**METHODS**			
Eligibility criteria	5	Specify the inclusion and exclusion criteria for the review and how studies were grouped for the syntheses.	Sections 2.2 and 2.7
Information sources	6	Specify all databases, registers, websites, organisations, reference lists and other sources searched or consulted to identify studies. Specify the date when each source was last searched or consulted.	Section 2.3
Search strategy	7	Present the full search strategies for all databases, registers and websites, including any filters and limits used.	Section 2.3; Table S2
Selection process	8	Specify the methods used to decide whether a study met the inclusion criteria of the review, including how many reviewers screened each record and each report retrieved, whether they worked independently, and if applicable, details of automation tools used in the process.	Section 2.4
Data collection process	9	Specify the methods used to collect data from reports, including how many reviewers collected data from each report, whether they worked independently, any processes for obtaining or confirming data from study investigators, and if applicable, details of automation tools used in the process.	Section 2.5
Data items	10a	List and define all outcomes for which data were sought. Specify whether all results that were compatible with each outcome domain in each study were sought (e.g. for all measures, time points, analyses), and if not, the methods used to decide which results to collect.	Sections 2.5 and 2.7
	10b	List and define all other variables for which data were sought (e.g. participant and intervention characteristics, funding sources). Describe any assumptions made about any missing or unclear information.	Section 2.5
Study risk of bias assessment	11	Specify the methods used to assess risk of bias in the included studies, including details of the tool(s) used, how many reviewers assessed each study and whether they worked independently, and if applicable, details of automation tools	Section 2.6; Table S3

Section and Topic	Item #	Checklist item	Location where item is reported
		used in the process.	
Effect measures	12	Specify for each outcome the effect measure(s) (e.g. risk ratio, mean difference) used in the synthesis or presentation of results.	Sections 2.7 and 2.8
Synthesis methods	13a	Describe the processes used to decide which studies were eligible for each synthesis (e.g. tabulating the study intervention characteristics and comparing against the planned groups for each synthesis (item #5)).	Section 2.7
	13b	Describe any methods required to prepare the data for presentation or synthesis, such as handling of missing summary statistics, or data conversions.	Sections 2.7 and 2.8
	13c	Describe any methods used to tabulate or visually display results of individual studies and syntheses.	Section 2.8; Table 1; Figures 2–4
	13d	Describe any methods used to synthesize results and provide a rationale for the choice(s). If meta-analysis was performed, describe the model(s), method(s) to identify the presence and extent of statistical heterogeneity, and software package(s) used.	Section 2.8
	13e	Describe any methods used to explore possible causes of heterogeneity among study results (e.g. subgroup analysis, meta-regression).	Section 2.8
	13f	Describe any sensitivity analyses conducted to assess robustness of the synthesized results.	Section 2.8
Reporting bias assessment	14	Describe any methods used to assess risk of bias due to missing results in a synthesis (arising from reporting biases).	Section 2.8
Certainty assessment	15	Describe any methods used to assess certainty (or confidence) in the body of evidence for an outcome.	Section 2.8 (GRADE)
**RESULTS**			
Study selection	16a	Describe the results of the search and selection process, from the number of records identified in the search to the number of studies included in the review, ideally using a flow diagram.	Section 3.1; Figure 1
	16b	Cite studies that might appear to meet the inclusion criteria, but which were excluded, and explain why they were excluded.	Section 3.1; Figure 1
Study characteristics	17	Cite each included study and present its characteristics.	Section 3.2; Table 1
Risk of bias in studies	18	Present assessments of risk of bias for each included study.	Section 3.3; Table 1; Table S3
Results of individual studies	19	For all outcomes, present, for each study: (a) summary statistics for each group (where appropriate) and (b) an effect estimate and its precision (e.g. confidence/credible interval), ideally using structured tables or plots.	Table 1; Figures 2–4

Section and Topic	Item #	Checklist item	Location where item is reported
Results of syntheses	20a	For each synthesis, briefly summarise the characteristics and risk of bias among contributing studies.	Sections 3.3 and 3.4
	20b	Present results of all statistical syntheses conducted. If meta-analysis was done, present for each the summary estimate and its precision (e.g. confidence/credible interval) and measures of statistical heterogeneity. If comparing groups, describe the direction of the effect.	Section 3.4; Figures 2–4
	20c	Present results of all investigations of possible causes of heterogeneity among study results.	Section 3.5; Table 2
	20d	Present results of all sensitivity analyses conducted to assess the robustness of the synthesized results.	Section 3.5; Table 2
Reporting biases	21	Present assessments of risk of bias due to missing results (arising from reporting biases) for each synthesis assessed.	Section 3.4; Table 2
Certainty of evidence	22	Present assessments of certainty (or confidence) in the body of evidence for each outcome assessed.	Section 3.4; Table 2 (GRADE)
**DISCUSSION**			
Discussion	23a	Provide a general interpretation of the results in the context of other evidence.	Sections 4.1–4.6
	23b	Discuss any limitations of the evidence included in the review.	Section 4.7
	23c	Discuss any limitations of the review processes used.	Section 4.7
	23d	Discuss implications of the results for practice, policy, and future research.	Section 4.8; Section 5
**OTHER INFORMATION**			
Registration and protocol	24a	Provide registration information for the review, including register name and registration number, or state that the review was not registered.	Section 2.1; Abstract
	24b	Indicate where the review protocol can be accessed, or state that a protocol was not prepared.	Section 2.1
	24c	Describe and explain any amendments to information provided at registration or in the protocol.	Section 2.1 (deviations from the protocol)
Support	25	Describe sources of financial or non-financial support for the review, and the role of the funders or sponsors in the review.	Funding statement
Competing interests	26	Declare any competing interests of review authors.	Conflicts of Interest statement

Section and Topic	Item #	Checklist item	Location where item is reported
Availability of data, code and other materials	27	Report which of the following are publicly available and where they can be found: template data collection forms; data extracted from included studies; data used for all analyses; analytic code; any other materials used in the review.	Data Availability Statement

From: Page MJ, McKenzie JE, Bossuyt PM, Boutron I, Hoffmann TC, Mulrow CD, et al. The PRISMA 2020 statement: an updated guideline for reporting systematic reviews. BMJ 2021;372:n71. doi: 10.1136/bmj.n71

For more information, visit: http://www.prisma-statement.org/

Table S2. Complete search strategies for each database.

Database (platform)	Date of search	Search strategy	Records
PubMed (MEDLINE)	July 2026	#1 "Burnout, Professional"[MeSH Terms] OR "Burnout, Psychological"[MeSH Terms] OR Burnout[Title/Abstract]<br>#2 "Emergency Medical Services"[MeSH Terms] OR "Emergency Medical Technicians"[MeSH Terms] OR "Prehospital Emergency Care"[Title/Abstract] OR "Ambulance Personnel"[Title/Abstract] OR Paramedics[Title/Abstract] OR "Emergency Medical Technicians"[Title/Abstract] OR "Out-of-Hospital Care"[Title/Abstract] OR "Prehospital Providers"[Title/Abstract]<br>#3 "Maslach Burnout Inventory"[Title/Abstract] OR MBI[Title/Abstract] OR "Copenhagen Burnout Inventory"[Title/Abstract] OR CBI[Title/Abstract]<br>#4 #1 AND #2 AND #3	180
CINAHL Complete (EBSCOhost)	July 2026	S1 (MH "Burnout, Professional") OR TI Burnout OR AB Burnout<br>S2 (MH "Emergency Medical Services+") OR (MH "Emergency Medical Technicians") OR TI ("Prehospital Emergency Care" OR "Ambulance Personnel" OR Paramedics OR "Emergency Medical Technicians" OR "Out-of-Hospital Care" OR "Prehospital Providers") OR AB ("Prehospital Emergency Care" OR "Ambulance Personnel" OR Paramedics OR "Emergency Medical Technicians" OR "Out-of-Hospital Care" OR "Prehospital Providers")<br>S3 TI ("Maslach Burnout Inventory" OR MBI OR "Copenhagen Burnout Inventory" OR CBI) OR AB ("Maslach Burnout Inventory" OR MBI OR "Copenhagen Burnout Inventory" OR CBI)<br>S4 S1 AND S2 AND S3	225
Scopus (Elsevier)	July 2026	TITLE-ABS-KEY (burnout OR "burnout syndrome") AND TITLE-ABS-KEY ("emergency medical services" OR "prehospital emergency care" OR "ambulance personnel" OR paramedics OR "emergency medical technicians" OR "out-of-hospital care" OR "prehospital providers") AND TITLE-ABS-KEY ("Maslach Burnout Inventory" OR mbi OR "Copenhagen Burnout Inventory" OR cbi)	106
Web of Science Core Collection (Clarivate)	July 2026	TS=(burnout OR "burnout syndrome") AND TS=("emergency medical services" OR "prehospital emergency care" OR "ambulance personnel" OR paramedics OR "emergency medical technicians" OR "out-of-hospital care" OR "prehospital providers") AND TS=("Maslach Burnout Inventory" OR MBI OR "Copenhagen Burnout Inventory" OR CBI)	110
LILACS (Virtual Health Library, BVS)	July 2026	(mh:"Burnout, Professional" OR burnout) AND (mh:"Emergency Medical Services" OR mh:"Emergency Medical Technicians" OR "prehospital emergency care" OR "ambulance personnel" OR paramedics OR "out-of-hospital care" OR "prehospital providers") AND ("Maslach Burnout Inventory" OR MBI OR "Copenhagen Burnout Inventory" OR CBI) AND db:("LILACS")	30
Total			651

No date, language or publication-type limits were applied in the database searches; language criteria (English, Spanish or Portuguese) were applied during screening. Backward (reference lists) and forward (citing articles identified with the "Cited by" functions of Web of Science and Scopus) citation searching of the included studies was also performed, and records were managed in Zotero. MeSH, Medical Subject Headings; MH, CINAHL Subject Heading; mh, DeCS/MeSH descriptor in the Virtual Health Library; TS, topic field (title, abstract, author keywords and Keywords Plus); TITLE-ABS-KEY, title, abstract and keywords.

Table S3. Risk of bias of the included studies assessed with the JBI Critical Appraisal Checklist for Studies Reporting Prevalence Data.

Study [ref]	Q1	Q2	Q3	Q4	Q5	Q6	Q7	Q8	Q9	Yes (/9)	Main issues
Bernaldo-De-Quirós et al., 2015 [17]	Y	Y	Y	Y	Y	Y	Y	Y	Y	9	Random sample; Raosoft sample size; response 89.5%
Coskun Cenk, 2019 [18]	Y	N	N	Y	U	Y	Y	Y	U	5	Web survey without randomization; sample below planned size (authors)
Deniz et al., 2016 [19]	Y	U	U	Y	U	U	Y	Y	U	4	Staff of one province; MBI version not specified
Crowe et al., 2020 [21]	Y	Y	Y	Y	Y	Y	Y	Y	Y	9	Statewide invitation (254/267 agencies); non-response analysis
Miller, 2026 [22]	Y	Y	N	Y	U	Y	Y	Y	U	6	Single station (n = 78); response 67.8%
Nabi-Amjad et al., 2023 [33]	Y	Y	Y	Y	U	Y	Y	U	U	6	Census of provincial staff; response rate not reported; inconsistent correlation (r = −0.553 in abstract vs. 0.62 in text)
Thielmann et al., 2024 [34]	Y	N	U	Y	U	Y	Y	Y	U	5	Online convenience sample; response rate not calculable
Sung & Lee, 2025 [35]	Y	U	Y	Y	U	Y	Y	Y	U	6	Sample size calculated (G*Power); all eligible invited via URL/QR; response rate not reported
Iorga et al., 2015 [36]	N	U	U	U	U	U	Y	U	U	1	Includes ambulance drivers; sampling, setting and burnout categories poorly described
Sánchez-Rodríguez et al., 2001 [37]	Y	U	N	Y	U	Y	Y	Y	U	5	73 respondents; denominator not reported
Koval et al., 2020 [38]	Y	N	Y	Y	Y	Y	Y	Y	Y	8	Convenience sample at training sessions; 94% scorable
Tunaligil et al., 2016 [39]	Y	Y	Y	Y	U	Y	Y	Y	U	7	All stations invited; Raosoft sample size; participation 55.4%
Avcı & Demirci, 2026 [40]	Y	U	Y	Y	U	Y	Y	Y	U	6	Sample size calculated; sampling procedure and response rate unclear
Wongtongkam, 2017 [41]	Y	N	N	Y	N	U	Y	Y	N	4	Response 10%; abbreviated 12-item MBI
Abareshi et al., 2022 [42]	Y	Y	Y	Y	U	N	Y	U	U	5	Random sample; sample-based tertile cut-offs with overlapping ranges
Liu et al., 2024 [43]	U	Y	Y	Y	U	Y	Y	Y	U	6	Census; 92% also work in hospital emergency departments; MBI-GS total score
Haruna et al., 2023 [44]	Y	Y	Y	Y	N	Y	Y	Y	N	7	Random selection of stations; response 21.8%
Kim et al., 2023 [45]	Y	Y	Y	Y	U	Y	Y	Y	U	7	All Seoul EMS providers invited; 51% participated

Study [ref]	Q1	Q2	Q3	Q4	Q5	Q6	Q7	Q8	Q9	Yes (/9)	Main issues
van der Ploeg & Kleber, 2003 [46]	Y	Y	U	Y	N	Y	Y	Y	N	6	Random selection of services; response 56% at baseline, 31% at follow-up
Roth et al., 2025 [47]	Y	N	Y	Y	U	Y	Y	Y	U	6	Same online EMSiG survey as [48]; response rate not calculable
Baier et al., 2018 [48]	Y	N	Y	Y	U	Y	Y	Y	U	6	Online recruitment via social media/journals; response rate not calculable
Luz et al., 2017 [49]	Y	Y	N	Y	Y	U	Y	Y	Y	7	32 of 40 staff (80%); Lautert cut-offs; drivers excluded from extraction
Rosales-Flores et al., 2022 [50]	U	N	N	Y	U	Y	Y	Y	U	4	Self-selected sample including 25% volunteers
Boland et al., 2018 [51]	U	Y	Y	Y	U	Y	Y	Y	U	6	Includes 19 dispatchers; response 54%
Amro et al., 2022 [53]	Y	N	Y	Y	N	Y	Y	Y	N	6	Convenience sample; response 28%
Vladič & Kren, 2021 [54]	Y	U	U	Y	U	Y	Y	Y	U	5	Sampling and response rate not clearly reported
Kukowski et al., 2016 [55]	Y	N	N	Y	Y	Y	Y	Y	Y	7	Convenience sample of 87 paramedics; response 96%
França et al., 2012 [56]	Y	Y	N	Y	Y	U	Y	U	Y	6	38 of 42 nurses (90%); non-standard cut-offs; reported % not consistent with n
Almutairi & El Mahalli, 2020 [57]	Y	Y	Y	Y	U	Y	Y	Y	U	7	Proportional allocation; G*Power sample size
Khatiban et al., 2015 [58]	Y	U	Y	Y	Y	Y	Y	U	Y	7	Consecutive sampling; response 92.2%; low PA category confirmed as PA ≤ 33 (high frequency of lack of PA)
Khashaba et al., 2014 [59]	Y	Y	U	Y	U	Y	Y	Y	U	6	Random selection of 140 EMRs; response rate not reported

JBI, Joanna Briggs Institute; Q, checklist question; Y, yes; N, no; U, unclear. Studies are listed by their reference number in the main text.

Q1. Was the sample frame appropriate to address the target population?

Q2. Were study participants sampled in an appropriate way?

Q3. Was the sample size adequate?

Q4. Were the study subjects and the setting described in detail?

Q5. Was the data analysis conducted with sufficient coverage of the identified sample?

Q6. Were valid methods used for the identification of the condition?

Q7. Was the condition measured in a standard, reliable way for all participants?

Q8. Was there appropriate statistical analysis?

Q9. Was the response rate adequate, and if not, was the low response rate managed appropriately?
